# Processing and Mechanical Properties of Highly Filled PP/GTR Compounds

**DOI:** 10.3390/ma15113799

**Published:** 2022-05-26

**Authors:** Artur Kościuszko, Dariusz Sykutera, Piotr Czyżewski, Stefan Hoyer, Lothar Kroll, Bogusław Szczupak

**Affiliations:** 1 Department of Manufacturing Techniques, Bydgoszcz University of Science and Technology, 85-796 Bydgoszcz, Poland; sykutera@pbs.edu.pl (D.S.); p.czyzewski@pbs.edu.pl (P.C.); 2 Department of Lightweight Structures and Polymer Technology (SLK), Chemnitz University of Technology, 09126 Chemnitz, Germany; stefan.hoyer@mb.tu-chemnitz.de (S.H.); lothar.kroll@mb.tu-chemnitz.de (L.K.); 3 Faculty of Information and Communication Technology, Wrocław University of Science and Technology, 50-372 Wrocław, Poland; boguslaw.szczupak@pwr.edu.pl

**Keywords:** rubber recycling, polypropylene, ground tire rubber, compounds, injection molding

## Abstract

Ground rubber from automobile tires is very difficult to recycle due to the cross-linking of the macromolecules and thus the lack of thermoplastic properties. The research consisted of assessing the processing possibility via the injection of highly filled PP/GTR compounds modified with 1.5 wt.% 2.5-dimethyl-2.5-di-(tert-butylperoxy)-hexane. GTR dosing ranged from 30 wt.% up to 90 wt.%. The evaluation of the processing properties of the obtained materials was carried out on the basis of the melt flow index test results and the signals recorded during processing by the injection molding by temperature and pressure sensors placed in the mold. The influence of the applied modifier on the changes in the mechanical properties of PP/GTR was determined with hardness, impact and static tensile tests. Moreover, thermal properties were obtained by the differential scanning calorimetry method. It has been found that it is possible to efficiently process compounds with high GTR content using injection molding. The presence of the filler allows to significantly reduce the cooling time in the injection mold and thus the time of the production cycle. It has been confirmed that 2.5-dimethyl-2.5-di-(tert-butylperoxy)-hexane modifies the rheological properties of PP and thus the PP/GTR composition. The lower viscosity of the matrix results in a more accurate bonding with the developed surface of the GTR grains, which results in better mechanical properties of the rubber-filled polypropylene.

## 1. Introduction

In a circular economy characterized by minimizing the use of raw materials and volume of waste, mechanical recycling plays an important role. Nowadays, more than 30 wt.% of the polymer post-consumer waste produced in Europe is mechanically recycled. Polypropylene (PP) is often a matrix of composites filled with natural fillers, ground cross-linked polymeric material, or minerals because it is one of the cheapest and most frequently used thermoplastics [1,2,3,4]. PP is used both in the form of homo-polypropylene and random and block copolymers with polyethylene. Filling inorganic or organic particles into the polypropylene matrix causes an increase in the apparent viscosity of the molten PP [5,6]. For this reason, the production of highly filled polyolefin composites requires high injection pressures, high clamping force, and high-level values of melt temperatures. Those parameters often cause an increase in the specific energy consumption (per cycle). A positive effect related to the filling of thermoplastics (particularly semi-crystalline ones) is the reduction of the shrinkage value [7,8]. In the case of many applications, the usually obtained increase in the stiffness of the composite material is also beneficial compared to the unfilled thermoplastic matrix [9].

The accumulation of used car tires is a global problem. Around 1.0–1.2 billion waste tires are produced every year, and the recycling industry processes only one-tenth of that number [10,11]. According to the latest EU directive, the mechanical recycling of waste tires is preferred [12]. Due to the cross-linked structure and elasticity of car tires, the process of grinding them consumes a lot of energy. The obtained recyclate can be used as a filler in cross-linked polymeric matrices [13,14,15,16]. The application of rubberized asphalt to construct pavements or the use of GTR as a modifier to the concrete are additional methods of recycled tire use [17,18,19,20]. There are also attempts to use rubber powder in additive technologies. Alkadi et al. used silane-modified rubber grains as a resin matrix filler in a 3D printing method [21].

In recent years, the possibility of mass use of GTR as a filler for thermoplastic polymers [22,23,24,25], including polypropylene [26,27,28], has been observed. Basso [29] described the effect of GTR grain size on the selected functional properties (hardness and roughness) of homo-polypropylene compounds. Compounds samples with max. A GTR content of 80 wt.% was achieved using injection molding. Higher content of the rubber fraction (90 wt.%) in the PP/GTR compounds was obtained using compression molding [30]. In injection molding or compression molding, the maximum process temperature should not exceed 200 °C. Higher temperatures can cause the thermal degradation of rubber grains.

Mujal-Rosas [31] found that the increase in GTR content in homo-polypropylene decreases the material stiffness, tensile strength, and impact strength. This is due to the specific elasticity properties of cross-linked rubber and its lower mechanical strength. More advantageous mechanical properties were obtained for compounds containing the smallest size of rubber grains (<200 µm). The beneficial effect of small grain size and large specific surface area on the mechanical properties has also been described by other authors [12,16,32]. As in the case of other fillers, using GTR as filler of PP matrix causes an increase in melt viscosity compared to unfilled PP; this was confirmed by Lima et al. [33]. Due to the limited compatibility between rubber particles and the polymeric matrix, the adhesion at the interface between the components improved. Da Silva [34] assessed the effect of PP/GTR blend compatibility on the structure, thermal and mechanical properties of the obtained materials. Polypropylene functionalized with maleic anhydride (PP-g-MAH) in an amount of 5 wt.% was used as a modifier, increasing the impact strength of the tested materials. Another method used to improve the adhesion between the GTR and the polypropylene matrix was the modification of rubber with concentrated sulfuric (VI) acid [35,36]. Treatment caused changes to the shape and surface of rubber grains. Increasing the specific surface caused better adhesion and increased the modulus of elasticity and tensile strength compared to materials with the same GTR content that had not been treated [37].

There was also research involving the modification of PP/GTR compounds using organic peroxides. Kim [38] investigated the effect of dicumyl peroxide and 2.5-dimethyl-2.5-di- (tert-butylperoxy) -hexane (DHBP) in the range from 0 to 1.5% wt.% on the properties of PP/GTR moldings by 70 wt.% of rubber filler content. Wagenknecht [39] investigated the effect of 2% DHBP dosing on the mechanical properties of PP random copolymer/GTR compounds with a filler content ranging from 30 wt.% up to 70 wt.%. This type of modification results in crosslinking of the filler with the matrix. Better adhesion at the interface causes increases in the tensile strength and elongation at the break of PP/GTR. It should be noted that the modification of GTR filled co-polypropylene with organic peroxides, in addition to the phenomena of cross-linking, may also result in a controlled degradation of macromolecules leading to an increase in the mass melt flow rate of the polymer. This was indicated in research published by Coutinho [40]. The introduction of DHBP in an amount of 0.6 wt.% into homo-polypropylene caused the MFR to increase from 3 to about 11 g/10 min. However, no publications describe the effect of the simultaneous modification of PP copolymer with GTR and DHBP on both the course of the injection molding process and the mechanical and rheological properties of moldings. The research conducted in this area focused mainly on assessing the effect of cross-linking between the matrix and the filler particles. Moreover, in investigations related to the modification of PP with GTR and DHBP, the GTR filling degree did not exceed 70 wt.%.

The aim of the paper was to investigate the effect of high GTR content on the processing and mechanical properties of compounds obtained by injection molding. The PP matrix was filled with GTR in the range of 30 to 90 wt.%. Rubber grains of about 500 µm were created by the precise grinding of rubber in a special knife mill. Additionally, the influence of the 2.5-dimethyl-2.5-di-(tert-butylperoxy)–hexane organic peroxide content, which was used as a flow modifier of the PP matrix, was investigated.

## 2. Materials and Methods

### 2.1. Materials

Commercial grade polypropylene with random copolymer Moplen RP 248R manufactured by LyondellBasell (Rotterdam, The Netherlands) was used as a matrix. This material was proposed for processing by injection molding. The mass flow rate (MFR) of Moplen RP 248R was 30 g/10 min (230 °C, 2.16 kg), the modulus of elasticity (*E*) and tensile stress at yield (*σ_y_*), as declared by the manufacturer, were 900 MPa and 22 MPa, respectively. Commercial random polypropylene copolymers are most often propylene-ethylene copolymers (usually 1–7% of ethylene content) in which the arrangement of the units is random.

In the investigations, pre-ground rubber from car tires supplied by PVP Triptis GmbH (Triptis, Germany) was used. The average rubber particle size was 7 mm. The GTR density was 1220 kg/m^3^. 2.5-Dimethyl-2.5-di-(tert-butylperoxy)-hexane (DHBP) in a polypropylene matrix, commercially available in the form of granules under the trade name PEROXAN HX 20 PP (Pergan GmbH, Bocholt, Germany), was used as the modifier. The content of organic peroxide in the granules was 20 wt.%. In the research work in the field of PP/GTR compound modification with the use of organic peroxides, apart from DHBP, dicumyl peroxide (DCP) was also used. However, the results of Kim’s research [38] indicate that more favorable mechanical properties of mixtures of PP with GTR can be obtained using DHBP; therefore, this type of modifier was used in the research.

### 2.2. Samples Preparation

**Figure 1:**
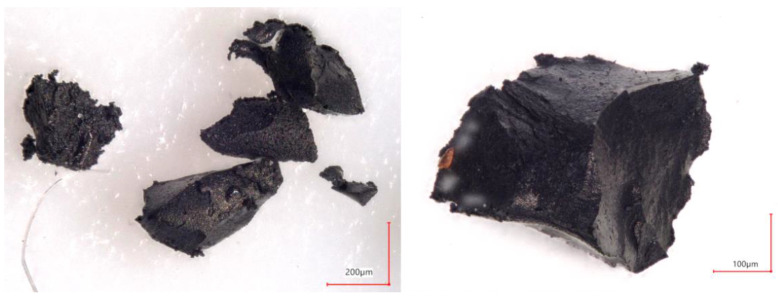
GTR particles after grinding process.

**Figure 2:**
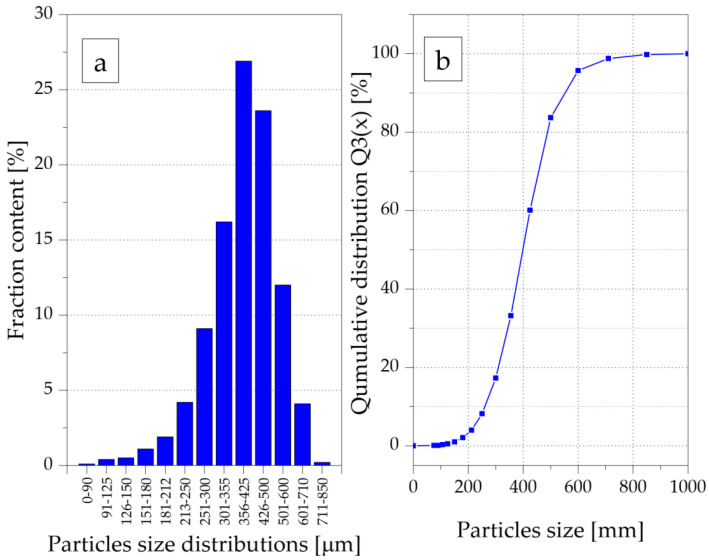
GTR filler size distribution obtained by CAMSIZER^®^ P4 (Retsch, Haan, Germany): histogram (**a**), qumulative curve (**b**).

The first stage of the test sample preparation consisted of GTR grinding into smaller particles. Rubber granulates with a grain size of about 7 mm were precisely ground to a grain fraction in the range of 100–600 µm using the original knife mill [12,32]. In the tests, two sieves with a mesh size of 500 µm were used, and the peripheral speed of the cutting edges of the movable knives was 14 m/s. The hyperboloid cutting method, described in previous publications [41], was used to grind the rubber. The rubber grains obtained by knife cutting were characterized by flat surfaces and sharp edges; this proves that the main reason for the disintegration of the granulate was because of the cutting with straight knife edges in the intersection gap of 0.1 mm between the cutting edges of the knives. In this way, rubber particles with an average size of 400 µm were obtained. Photographs of exemplary GTR particles after grinding are shown in Figure 1. Grains ranging from 300 to 500 µm were dominant in the obtained powder (Figure 2a) and about 90% were smaller than 500 µm (Figure 2b).

**Figure 3:**
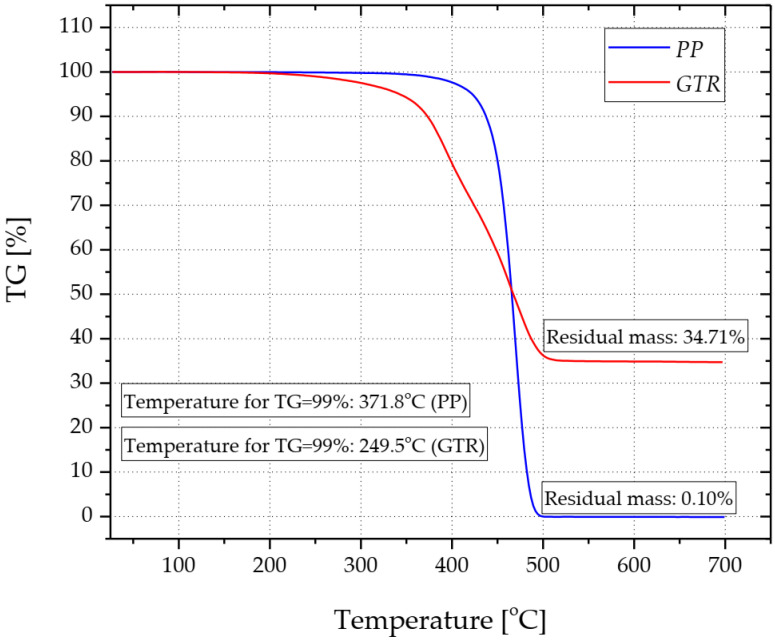
Thermal stability of PP and GTR determined by TG 209 Libra (Netzsch, Selb, Germany).

Subsequently, the GTR and PP granules after pre-mixing were compounded using a single-screw extruder manufactured by the Institute of Plastics Processing in Torun (Torun, Poland). The screw diameter was 25 mm, while the ratio of the screw length to its diameter (*L*/*D*) was 35 mm. The temperature in the individual zones of the plasticizing system was, respectively, 190 °C (in the head), 190 °C (in the metering zone), 175 °C (in the compression zone) and 135 °C (in the feed zone). Despite the fact that the polypropylene copolymer can be processed at a higher temperature, the maximum temperature of the plasticizing system did not exceed 190 °C. The aim was to limit the processes of thermal decomposition of the rubber filler, which is characterized by significantly lower thermal stability compared to the thermoplastic matrix. Figure 3 presents exemplary thermogravimetric (TG) curves determined for the Moplen RP 248R copolymer and GTR. The temperature at which the mass of the sample during the thermogravimetric test is reduced by 1% was assumed to be the thermal stability Moreover, on the basis of thermogravimetric tests, it was determined that the content of mineral filler in the rubber was about 35 wt.%. In the case of PP, residual mass equal to 0.1% indicates that the thermoplastic polymer was unfilled.

Seven polypropylene compositions containing from 30 to 90 wt.% GTR were prepared (first series). The second series included PP/GTR compounds, which were modified with 2.5-dimethyl-2.5-di-(tert-butylperoxy)-hexane to the amount of 1.5 wt.%. Moreover, copolymer reference samples were prepared, one of which was modified with organic peroxide. The research work focused on highly filled PP compounds with GTR, as processing low-filled materials by injection molding is not difficult. Moreover, tires are a mass-scale waste; therefore, it is practical to research highly filled compounds. A list of all prepared PP/GTR compounds is presented in Table 1.

**Figure 4:**
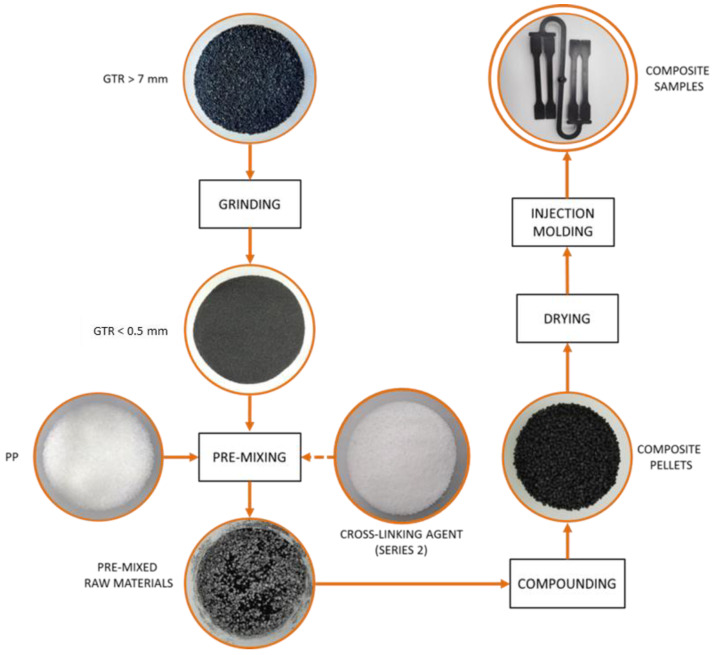
Diagram of the preparation of PP/GTR samples.

The samples for mechanical properties investigations were made by injection molding using the Engel e-victory 110 hybrid injection molding machine (Schwertberg, Austria). A four-cavity injection mold, allowing for the production of universal A-type test samples with dimensions corresponding to the ISO 3167 standard was used. The temperature of individual zones of the plasticizing system was 190 °C (nozzle), 190 °C, 170 °C, 140 °C and 40 °C at the feed zone. The temperature of injection mold, injection rate, holding pressure and holding time were, respectively, 50 °C, 100 cm^3^/s, 22 MPa and 21 s. Immediately before the injection molding, all compounds were dried at 110 °C for 2 h. For this purpose, a FED 115 dryer by Binder (Tuttlingen, Germany) was used. The diagram of the preparation process of polypropylene compounds filled with GTR is shown in Figure 4.

### 2.3. Rheological Properties Measurements

The investigation of the rheological properties of PP/GTR consisted in determining their volume-melt flow rate (MVR). The tests were carried out with the use of an Aflow plastometer by Zwick/Roell (Ulm, Germany). The measurements were carried out at the temperature of 190 °C, with the test load weight equal to 10 kg. The diameter and length of the capillary were 2.095 mm and 8 mm, respectively. The mass of the test load was selected in such a way that it was possible to determine the MVR for two series of PP/GTR blends under the same conditions. The test temperature (190 °C) was lower compared to the unfilled polypropylene commonly used for measurements (230 °C) which results from an attempt to reduce the intensity of rubber decomposition processes at high temperatures.

**Figure 5:**
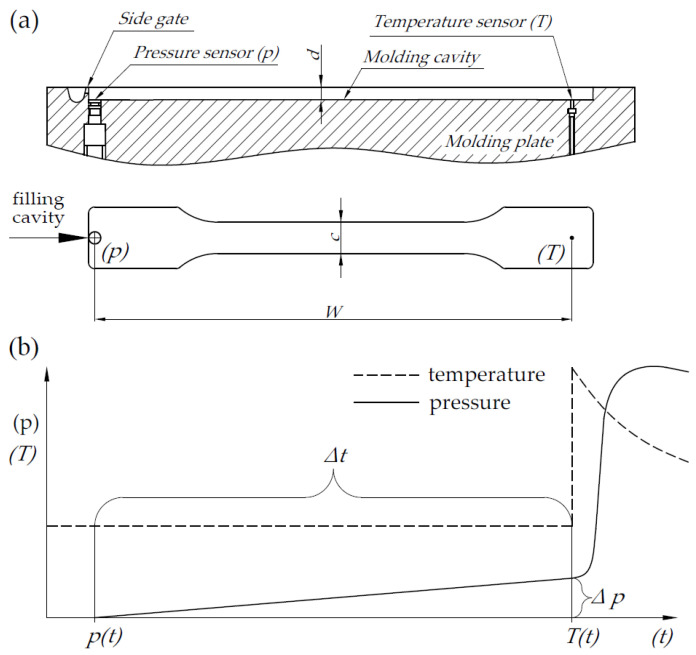
Method of determining the apparent viscosity during injection molding: placing of sensors in the mold cavity (**a**), changes of pressure and temperature in the mold cavity (**b**).

In addition, tests of the rheological properties of the obtained compositions were carried out under processing conditions by the injection method (during test samples preparation). Figure 5 shows the location of pressure and temperature sensors along with the characteristic dimensions of the molding cavity (Figure 5a) and the principle of determining the apparent viscosity in the injection mold by registering pressure and temperature changes while filling the mold cavity (Figure 5b).

The measuring system starts recording pressure and temperature each time the injection cycle is started (injection mold closure). The actual measurement of Δ*p* was made from the first increase in pressure (melt contact with the sensors) until the signal was received from the temperature sensor (Δ*T*). The obtained values of Δ*T* and Δ*p* are necessary to determine the value of the shear rate ($\dot{\gamma }$), shear stress (*τ*) or apparent viscosity (*η_a_*). The formula for the determination of shear stress value is:

(1) $$\tau_{i}=\frac{\Delta p\cdot d}{2\cdot W}$$

where Δ*p*—is the pressure increase between signals from pressure and temperature sensors, *d*—is the mold cavity depth, and *W*—is the distance between sensors in the mold cavity (Figure 5a). Shear rate values were calculated from the formula:

(2) $$\dot{\gamma_{i}}=\frac{6\cdot Q_{i}}{c\cdot d^{2}}$$

where *Q_i_*—is the flow rate of the polymer melt flow in the mold cavity, and *c*—is the width of the cavity mold. The flow rate of the polymer melt in the mold cavity was calculated by equation:

(3) $$Q_{i}=\frac{W\cdot c\cdot d}{\Delta t}$$

where Δ*t*—is the time the polymer melt needs to run a distance between the sensors. The formula for the shear rate, taking into account the flow rate relationship takes the form:

(4) $$\dot{\gamma_{i}}=\frac{6\cdot W}{\Delta t\cdot d}\;$$

The apparent viscosity, which is the ratio of shear stress to shear rate, can be described by the following equation:

(5) $$\eta_{a}=\frac{\tau }{\dot{\gamma }}=\frac{\Delta p\cdot \Delta t\cdot d^{2}}{12W^{2}}\;$$

### 2.4. FTIR Analysis of the Compounds Chemical Composition

In order to assess the possible occurrence of chemical transformations other than the expected selective cutting of PP chains during the processing of PP/GTR compounds with organic peroxide, spectrophotometric tests were carried out. Measurements were made using an INVENIO R infrared spectrophotometer by Bruker (Ettlingen, Germany) equipped with an ATR attachment with a diamond crystal. During the research, 128 scans were performed for each sample with a resolution of 4 cm^−1^ in the wavenumber range of 3100 to 400 cm^−1^. Measurements were made for samples of ground GTR, PP and PP-P and for chosen samples of PP/GTR compounds. During the tests, the core part of the samples was scanned in 15 different places due to the heterophasic composition of the samples containing GTR.

### 2.5. Measurements of Thermal Properties

Measurements of thermal properties of PP compounds filled with ground rubber from car tires were carried out using the differential scanning calorimetry (DSC) method using the DSC 214 Polyma apparatus by Netzsch (Selb, Germany). The 8–10 mg samples were heated to 190 °C under nitrogen to erase the thermal processing history. After two minutes of exposure to the set temperature, the samples were cooled to 25 °C and after another two minutes, it was reheated to 190 °C. The heating and cooling rate was 10 °C/min.

Based on the determined values of the melting enthalpy (Δ*H*) of unfilled PP and PP-P samples, the theoretical course of changes in the Δ*H* of PP/GTR and PP/GTR-P samples with different rubber filler content were determined. The theoretical values of the melting enthalpy of compounds (Δ*H_ti_*) were calculated from the following formula:

(6) $$\Delta H_{ti\;}=\omega_{i}\cdot \Delta H_{100\%}\;\;$$

where *ω_i_* is the mass fraction of the assumed filler content in the compound, and Δ*H*_100%_ is the determined enthalpy of melting of the unfilled matrix. Moreover, the values of the melting enthalpy of materials determined during calorimetric tests were used to assess the real GTR content in the obtained materials (*C_pi_*) calculated from the following formula:

(7) $$Cp_{i}=\left(1-\frac{\Delta H_{i}}{\Delta H_{100\%}}\right)\cdot 100\%\;\;\;$$

where the symbol Δ*H_i_* denotes the melting enthalpy of the sample determined during calorimetric tests.

### 2.6. Measurements of Mechanical Properties

The hardness tests of the PP compounds filled with GTR were performed using the Shore D method (ShD). The Shore D scale was used in the research because it enables the determination of the hardness of both thermoplastics (unfilled copolymer) and elastomers (highly filled PP/GTR compounds). The measurements were made using a digital hardness tester by Zwick/Roell (Ulm, Germany). The tests of each measurement series were carried out on 10 samples at the temperature of 23 °C.

The mechanical properties were also determined by tensile test according to the standard ISO 527-1 using a universal testing machine Z030 Zwick/Roell (Ulm, Germany) equipped with a measuring head of a nominal load of 30 kN and mechanical extensometer. The speed during the measurements of the elastic modulus was 1 mm/min. The next stage of the measurements was carried out at a speed of 50 mm/min until the samples were broken. The tests were performed at 23 °C for 10 samples from each measurement series.

The Charpy impact strength of samples was determined according to the standard ISO 179-1 using the HIT50P pendulum impact tester by Zwick/Roell (Ulm, Germany) with a pendulum of 5 J. The samples were rectangular with dimensions of 80 mm × 10 mm × 4 mm. The impact took place on the shorter edge of the samples. The tests were performed at 23 °C for 10 samples from each measurement series. Due to the heterophasic composition of the materials, the measurements were carried out using samples without a notch, even though not all samples broke. Cutting the notch would remove the sample’s skin layer, which has a lower GTR content compared to the core layer. Dynamic thermomechanical analysis (DMA) was carried out using the high-force DMA GABO Elexor apparatus by Netzsach (Selb, Germany). Tests in the tensile mode were carried out on samples with a rectangular shape (80 mm × 10 mm × 4 mm). The samples were heated from −75 to 50 °C at a rate of 2 °C/min. The deformation strain was 0.1% and the frequency was 10 Hz.

### 2.7. Measurements of Morphology

The structure of PP/GTR was investigated using the Keynece VHX-7000 digital microscope (Osaka, Japan). The optical device was equipped with a VH-Z100R lens that allows microscopic observations at a magnification of 100× to 1000×. The investigations were carried out for the PP/GTR moldings from two series with a filler content of 70 wt.%, and 90 wt.%, respectively. The cross-sections were obtained with a slow-speed saw ISOMET (Buehler, Esslingen, Germany) with a diamond blade. In order to evaluate the adhesion between the matrix and the rubber particles, the fractures of the PP/GTR50 and PP/GTR50-P samples after the tensile test were assessed.

## 3. Results

### 3.1. Rheological Properties

**Figure 6:**
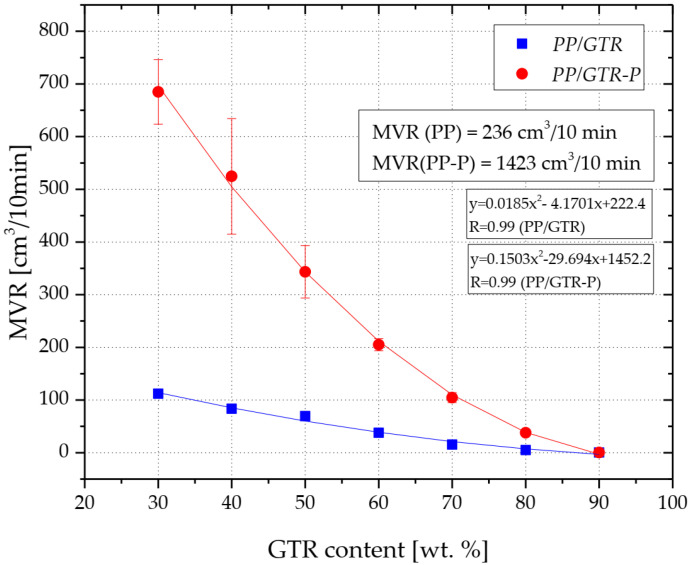
Influence of GTR content on MVR of PP/GTR compounds.

The increase in the content of GTR in the copolymer matrix results in a gradual reduction of the volume-flow rate (MVR) of the obtained compounds (Figure 6). The MVR value of unfilled PP was about 235 cm^3^/10 min. Introducing 70 wt.% of GTR into the thermoplastic matrix resulted in a reduction of MVR of over 90% (15.5 cm^3^/10 min). The PP/GTR90 was characterized by the value of the volume-melt flow rate equal to only 0.5 cm^3^/10 min, which was due to only 10% of the mass of the plasticized phase in the tested material. Edogage [30] also recorded the decrease in the melt flow index value due to the increase in GTR content in homo-polypropylene. However, these tests were performed under standard conditions for PP (230 °C, 2.16 kg), and PP had a clearly lower MFI than the matrix used in the present research work. Therefore, the test results cannot be compared with each other. It is also worth noting that the low MVR values of the composites with the highest GTR filling were recorded at a relatively large mass of load (10 kg) which may indicate the limited possibilities of using this type of compound in the injection technology. This motivates the need to carry out rheological tests directly in the processing tool.

The effect of using organic peroxide (2.5-dimethyl-2.5-di-(tert-butylperoxy)-hexane) for PP/GTR modification is a significant increase in the MVR of unfilled polypropylene and compounds with the lowest ground rubber content. For example, the MVR of the organic peroxide modified copolymer reached over 1400 cm^3^/10 min, while the MVR of the 70 wt.% GTR composition was over 90% (105 cm^3^/10 min) lower compared to the unfilled matrix. The MVR of the PP/GTR90-P (0.4 cm^3^/10 min) was slightly lower compared to the value recorded for the composition with the same filler content, which was not modified with DHBP (PP/GTR90). The MVR increase of the PP/GTR-P is due to the reactive extrusion of the compounds with organic peroxide. This phenomenon is due to the selective degradation of the propylene segments in the macromolecules, initiated by free radicals resulting from the thermal decomposition of the organic peroxide. This process leads to a decrease in the average molecular weight of polypropylene, which was published by Berzin for both the homopolymer and the copolymer [42]. Reactive extrusion of PP is commonly used in industry for the production of various types of PP with the desired rheological and mechanical properties [43].

**Figure 7:**
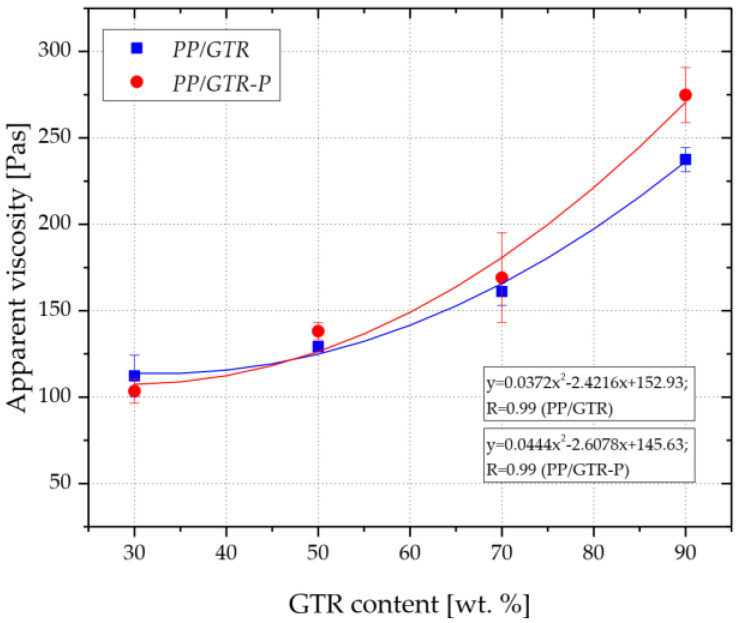
Influence of GTR content on the apparent viscosity of PP/GTR compounds.

The increase in the fluidity of the polypropylene copolymer due to the action of the organic peroxide is also confirmed by the results of the apparent viscosity test, which was determined during the filling of the injection mold cavity (Figure 7). The measurements were carried out for an injection speed equal to 125 cm^3^/s, which for the applied geometry of the injection mold, corresponded to a shear rate ranging from 1200 to 1500 L/s (Table 2). The average viscosity of the PP reached a value of 68 Pa·s, while a value of less than 20 Pa·s was recorded for the peroxide-modified PP (PP-P). Along with the increase in the filler content, an increase in the apparent viscosity of the materials was observed in the range of 100 Pa·s to about 250 Pa·s for both tested series. Starting from filling equally to 50 wt.%, higher values of apparent viscosity were recorded for compositions modified with organic peroxide. For example, the mean of the recorded *η_a_* values of the PP/GTR90-P was 275 Pa·s, while for the PP/GTR90 it was 237 Pa·s. It can, therefore, be concluded that the selective degradation of macromolecules, which results in an increase in MVR, results in a decrease in the apparent viscosity of unfilled matrix and PP/GTR compounds (GTR content less than 90 wt.%) only at low shear rates. At the higher values of shear rates by injection molding and at the high filling of PP matrix (from 50 wt.%), the effect of the apparent viscosity decreasing was not observed. The higher apparent viscosity of PP/GTR-P at high filling degrees GTR compared to PP/GTR is due to the increase in share stress during the flow of the compound in the cavity. The decrease in the viscosity of the PP melt in the PP/GTR-P mixture is most likely the result of the increased mechanical interaction (interlayer friction) of the GTR grains.

**Figure 8:**
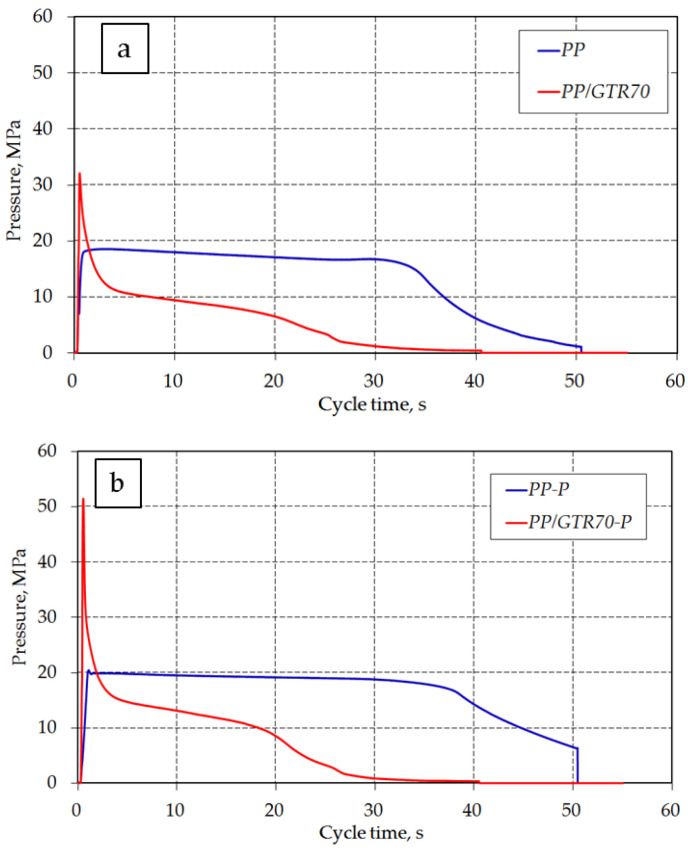
Pressure curves in the mold cavity for pure polypropylene and polypropylene filled with 70% GTR: (**a**) without DHBP, (**b**) addition of 1.5% DHBP.

The influence of GTR content and modifying DHBP on the changes in the rheological parameters of the PP compounds can also be observed during the sample preparation in the injection molding process. A significant reduction of filling pressure in the plasticizing unit for unfilled DHBP-modified PP was noted (Table 3). This effect results from the selective decomposition of the copolymer macromolecules due to the action of the organic peroxide. As a result of the reduction of the melt flow resistance, the injection pressure was reduced from 36.9 MPa (PP) to 8.0 MPa (PP-P). The addition of DHBP also extended the sealing time in the cavity gates and holding pressure (see Figure 8—blue curve). The addition of GTR powder to the PP matrix increases the melt flow resistance, and more significantly, the content of the rubber filler. Based on the recorded injection pressure values in the plasticizing unit of the injection molding machine and the injection mold, for the PP/GTR 90 wt.%, a significant increase in this parameter was observed in the zones mentioned above by 250% and 230%, respectively (Table 3). For PP/GTR compounds containing DHBP, additional flow resistance was recorded. The most significant increase in injection pressure (about 30%) was noted for PP/GTR50-P and PP/GTR70-P compared to materials with the same amount of GTR unmodified DHBP. An interesting effect was observed for the compound containing the maximum rubber content (PP/GTR90). The interlayer friction has a dominant effect on its flowability. The shear stresses occurring in the gate have reduced this effect. It was confirmed by slight changes in the cavity pressure for the PP/GTR90 melt. The addition of DHBP did not significantly affect the observed changes.

Figure 8 shows examples of pressure changes in the mold cavity at the injection speed of 125 mm/s for pure polypropylene and polypropylene filled with 70 wt.% GTR. The curves show the effect of DHBP addition on the pressure change in the molding cavity. The addition of GTR to the PP matrix also changes the pressure curve in the mold cavity (Figure 8). In the case of unfilled PP, the pressure curve characteristic for semi-crystalline thermoplastic polymers can be observed (visible constant pressure value during the holding phase). During the holding phase, a decrease in the pressure after the filling phase for PP/GTR compounds was observed. This effect can be noted during the injection molding process for highly filled compounds. Sykutera et al. observed a similar effect in microcellular injection molding investigations of semi-crystalline reinforced thermoplastic materials [44,45]. The lower content of PP in the composition results in faster solidification of the material in the mold cavity, as evidenced by changes in the pressure curve for PP/GTR (red color). Despite the constant pressure profile of the injection molding machine, the value of this parameter in the mold cavity was reduced to a minimum after 40 s of cycle time. The described effect is advantageous for economic and ecological criteria. It is possible to produce highly filled compounds PP/GTR in a shortened cycle time.

### 3.2. FT-IR Analysis

**Figure 9:**
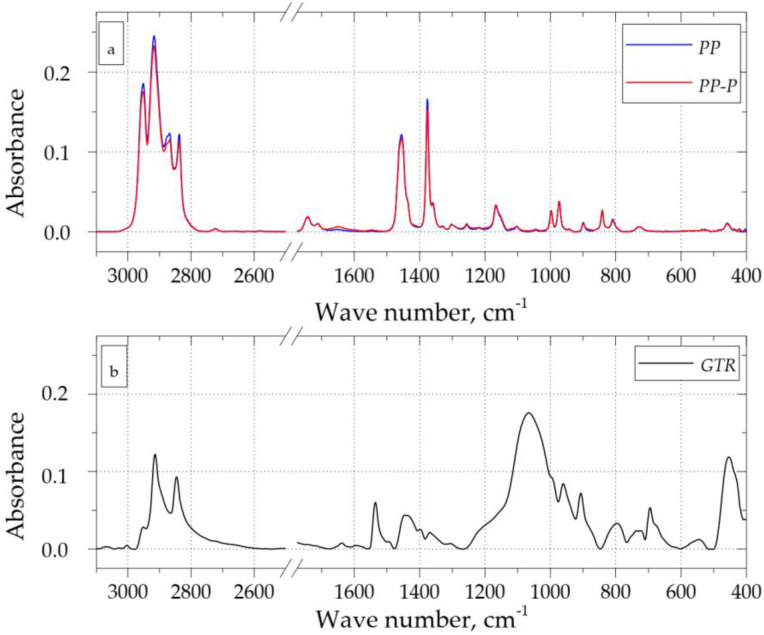
FT-IR spectra of PP, PP-P (**a**) and GTR samples (**b**).

According to Wagenknecht [39], during the reactive extrusion of a PP/GTR composition with peroxide, in addition to the undesirable selective cutting of macromolecules, the desired cross-linking process also takes place simultaneously. The ratio between these two phenomena is determined by the filler content and the parameters of the extrusion process. A similar mechanism for the reactive extrusion of this type of compound was proposed by Yoon [46]. On the other hand, Rooj [47] indicates that the effect of organic peroxide on natural rubber may lead to its devulcanization. Figure 9a shows the FTIR spectra recorded for PP and PP-P, which are very similar to each other. The lack of additional bands on the spectrum recorded for PP-P and the lack of a clear change in the intensity of the bands compared to the unmodified PP may indicate that during the reactive extrusion of the random PP copolymer, the dominant transformation was the selective cutting of macromolecules, which resulted in the changes in the rheological properties described above. Figure 9b shows the FT-IR spectrum of the ground rubber from car tires, which was used in the tests. For example, the area in the range 1500–800 cm^−1^ includes the absorbent bands of the elastomeric components. The 556 cm^−1^ band is assigned to the S-S bond and the 1538 cm^−1^ band to the C-S bond.

**Figure 10:**
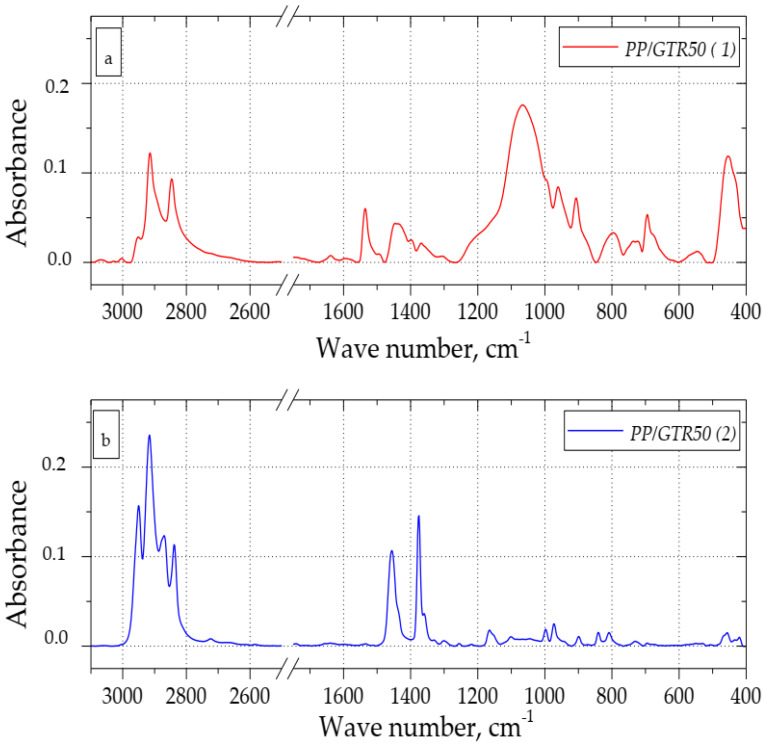
FT-IR spectra of PP/GTR50: sample 1 (**a**), sample 2 (**b**).

Figure 10a,b show the FT-IR spectra recorded during two measurements of the PP/GTR50 sample. The course of the spectrum recorded during the first measurement is similar to that recorded for pure GTR. The spectrum recorded for the second measurement corresponds with the bands characteristic of a random PP copolymer. This effect results from the heterophasic composition of the tested materials, in which the dimensions of individual rubber particles may exceed 500 µm. Moreover, it should be noted that the IR radiation only penetrates the sample to a depth of a few micrometers. Therefore, when performing spectrophotometric measurements of this type of material, it is uncertain what area of the sample has been scanned, i.e., whether it is the GTR grain, PP matrix, or the interfacial layer. The specificity of the PP/GTR samples and the specificity of the FT-IR spectrophotometry test with the ATR attachment mean that the correct analysis of the results and the assessment of the structure of this type of material may be difficult. The specificity of the PP/GTR samples and the specificity of the FT-IR spectrophotometric test with the ATR attachment make the interpretation of test results more difficult and so it should be based on the analysis of a significant number of spectra.

**Figure 11:**
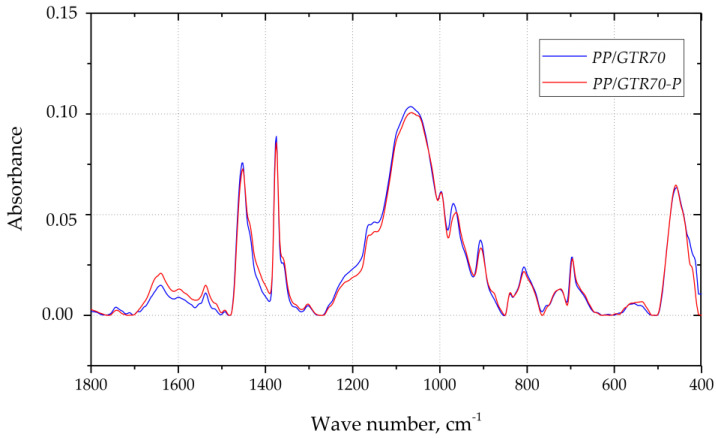
FT-IR spectra of interphase area recorded for PP/GTR70 (blue) and PP/GTR70-P (red).

Chosen FTIR spectra recorded during the tests of PP/GTR70 and PP/GTR70-P samples are shown in Figure 11. The bands in the range from 1500 cm^−1^ to 800 cm^−1^ indicate that the scanned area contains elastomeric components, and the intense bands present at the wavenumbers of 1456 cm^−1^ and 1376 cm^−1^, confirm that the examined area of the sample also contained PP. The similar intensity of the 556 cm^−1^ band for both spectra indicates that during the reactive extrusion there was no breakdown of the C-C bonds. The reactive extrusion of the tested compositions, therefore, does not lead to a devulcanization process. The lack of additional bands on the PP/GTR70-P spectrum and the lack of change in the intensity of the remaining bands may indicate that during the processing of PP with GTR and organic peroxide, there was also no cross-linking of the matrix with the filler, as indicated by Wagenknecht [39]. Thus, the dominant chemical process during the reactive random extrusion of PP-GTR copolymers is the process of selectively cutting matrix macromolecules.

### 3.3. Thermal Properties

**Figure 12:**
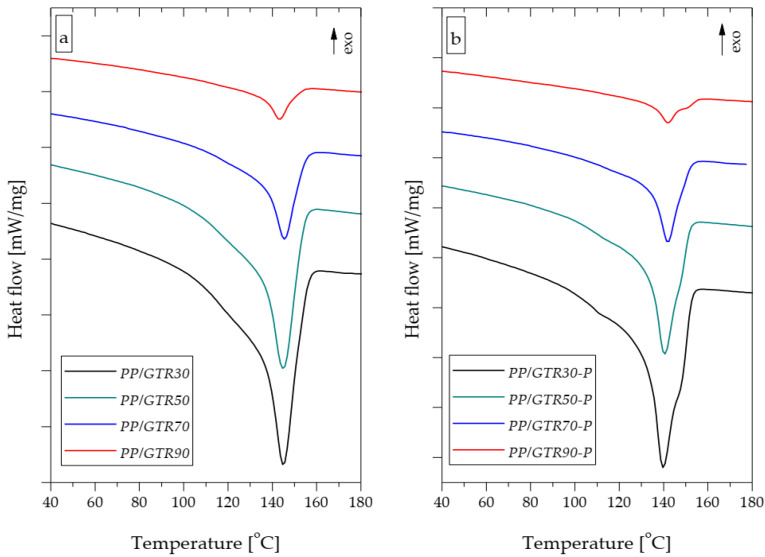
DSC melting curves of PP/GTR (**a**) and PP/GTR-P (**b**) compounds.

**Figure 13:**
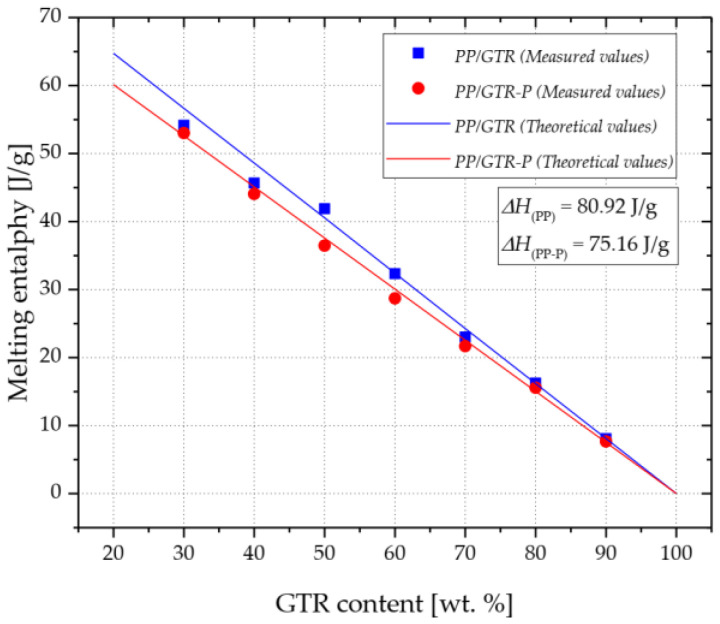
Theoretical and measured values of melting enthalpy of PP/GTR and PP/GTR-P.

Figure 12a,b shows the DSC melting curves of polypropylene compounds with a GTR content of 30%, 50%, 70% and 90 wt.%, respectively. With the increase in the GTR content in the material, a gradual decrease in the size of the endothermic melting peak is observed, which results from the decreasing amount of semi-crystalline, thermoplastic matrix. For example, the melting enthalpy of the PP/GTR30 sample was 53.44 J/g, while in the case of the PP/GTR90 composition, the value was recorded at 8.20 J/g (Figure 13). For the PP/GTR30-P and PP/GTR90-P compounds, the recorded values were 52.13 J/g and 7.76 J/g, respectively. The values of the melting enthalpy of the samples from the PP/GTR-P series were slightly lower compared to the samples from the PP/GTR series with the same rubber filler content. A similar effect was observed with the unfilled polypropylene samples. The melting enthalpy of PP-P was 75.16 J/g, while for the PP sample, the value was determined to be 80.92 J/g. The recorded differences most probably result from the selective degradation of the polypropylene matrix.

The melting enthalpy determined by calorimetric tests of samples was compared to the theoretical values of the enthalpy determined by measurements of the melting enthalpy of unfilled PP and PP-P samples (Figure 13). It was found that the values of the melting enthalpy determined for samples of PP/GTR and PP/GTR-P compounds with different contents of rubber filler in the material are similar to the calculated theoretical values of this parameter. The greatest differences were observed in the case of PP/GTR30 and PP/GTR40 samples, which indicates differences between the assumed and real rubber filler content in the tested samples. The PP/GTR30 compound was characterized by being less than 4 percentage points higher than the assumed rubber content, and in the case of the PP/GTR50 composition, the filler content was over 2.5 percentage points lower (Table 4). The determined differences between the real content of the filler in the composition and the assumed values may be the result of the specificity of the applied technologies for mixing the components and the preparation of test samples. Another reason may be the specificity of the calorimetric test, in which the mass of the tested sample was about 10 mg, i.e., about 0.015% of the weight of the moldings made of PP/GTR50.

The introduction of GTR into the polypropylene matrix resulted in a reduction of its crystallization temperature (*T_c_*) for both series of compounds (Table 4). The crystallization temperature of the PP and PP-P samples was equal to 119.5 °C and 119 °C, respectively, and the *T_c_* of the mixtures filled with ground rubber was over 10 °C lower. For example, the *T_c_* of the PP-GTR50 and PP/GTR50-P samples were 107.3 °C and 104.5 °C, respectively. This phenomenon is disadvantageous for application reasons; a longer residence time of the material in the injection mold during the cooling phase may be a consequence. Moreover, the presence of GTR in the matrix resulted in the reduction of the melting point (*T_m_*) of the polypropylene by about 5 °C. However, it should be noted that the *T_m_* of the PP-P sample was 4 °C lower (164 °C) compared to unmodified PP (150 °C). This effect can be explained by the shortening of the macromolecules of the copolymer matrix.

### 3.4. Mechanical Properties

**Figure 14:**
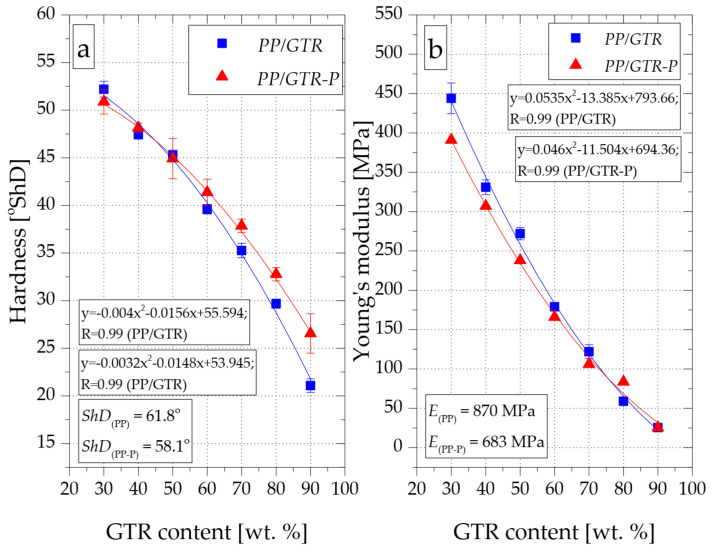
Influence of GTR content on hardness (**a**) and Young’s modulus (**b**) of PP/GTR compounds.

The introduction of GTR to the PP matrix results in a gradual decrease in the hardness of materials along with an increase in the mass fraction of the filler (Figure 14a). The hardness of the PP/GTR30 reached a value of about 52° ShD, while in the case of PP/GTR90 it was only 21° ShD, which in the Shore A scale corresponded to approximately 72° ShA. The hardness of the compound with the highest GTR content was about 65% lower compared to the unfilled matrix (62° ShD). Modification of PP with the use of organic peroxide (PP-P) resulted in a decrease in its hardness to about 58° ShD. A slightly lower hardness (51° ShD) compared to PP/GTR30 (52° ShD) was also recorded for the PP/GTR30-P composition. This effect is due to the selective chain degradation of the polypropylene random copolymer. However, compositions modified with an organic peroxide with a filler content greater than 50 wt.% were characterized by significantly higher hardness. For example, the hardness of the PP/GTR90-P was 26.5° ShD, i.e., it was about 20% higher compared to PP/GTR90. This effect is most likely related to the more accurate surrounding of the individual GTR grains by a less viscous matrix. The hardness of all obtained PP/GTR compounds is about 20° ShD lower compared to the hardness of samples obtained by Elenien et al. [35]. Additionally, the PP compound with 80 wt.% GTR content obtained by Basso had a higher hardness measured on the Shore D scale [29] compared to the PP/GTR80 obtained in the present study; this is probably from the use of homo-polypropylene by those researchers, which is characterized by higher hardness when used as the matrix of the compounds.

Selective degradation of the polymer chains of the thermoplastic matrix as a result of the introduction of 1.5 wt. 2.5-dimethyl-2.5-di-(tert-butylperoxy)-hexane also resulted in a decrease in the *E* value (Figure 14b). The modulus of elasticity of unmodified PP was 870 MPa, while for PP-P the recorded value was about 20% lower (683 MPa). The introduction of the GTR to PP matrix for both series of materials (PP/GTR and PP/GTR-P) resulted in a reduction of *E* along with an increase in the filler content. At the same time, a reduction in the difference in the value of Young’s modulus between the compositions of both series with the same filler content was observed. The tensile modulus of the composition with the highest GTR content (PP/GTR90 and PP/GTR90-P) was about 25 MPa in both cases, 97% less compared to the unmodified matrix.

**Figure 15:**
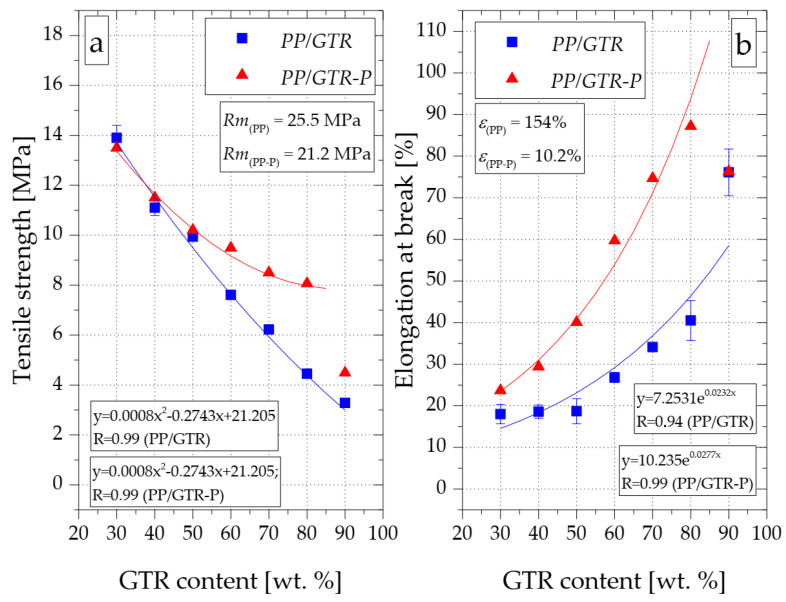
Influence of GTR content on tensile strength (**a**) and elongation at break (**b**) of PP/GTR compounds.

Figure 15a shows the relationship between the GTR content and the registered tensile strength (*Rm*) of the tested compounds. The tensile strength of the unfilled random copolymer with organic peroxide (PP-P) was over 15% lower (21.2 MPa) compared to the unmodified polymer (25.5 MPa). The introduction of GTR to both PP and PP-P resulted in a gradual decrease in the *Rm* value of the obtained materials. However, the dynamics of changes in tensile strength in the case of the PP/GTR-P series were clearly lower than in the case of the compositions not modified with the organic peroxide. For example, the tensile strength of the PP/GTR70 was 6.2 MPa, while PP/GTR70-P was found to be 35% higher (8.5 MPa). The results may indicate an increase in the adhesion between the matrix and GTR grains, which is the result of a more accurate surrounding of the individual GTR grains by the thermoplastic matrix. The mechanical properties of the PP/GTR, which have been modified with the use of organic peroxide, are determined by the processes of cutting macromolecules of the thermoplastic matrix and connecting the matrix with the filler particles. Selective degradation of the matrix results in decreasing its mechanical properties. The effect of the increase in interactions between the matrix and the elastomeric phase causes an increase in tensile strength. Moreover, the *Rm* results of compounds with 70% GTR content in the matrix are similar to the values obtained by Kim et al. [38] for compounds of similar compositions but clearly, it is less favorable than those obtained by Wagenknecht [39]. In this case, the highest recorded tensile stress of the compound with 70% GTR content was over 12 MPa. The *Rm* results of the PP/GTR90 were close to the tensile strength results by Egodage [30] for samples made by compression molding, despite the fact that in the research, PP waste was used as the matrix for the compounds, with different properties compared to the random copolymer. Therefore, it can be concluded that with a high filling degree of the compound, the *Rm* value is determined primarily by the GTR properties. It is also worth paying attention to the rapid decrease of *Rm* in the case of increasing the share of GTR in the compound to 90 wt.% (PP/GTR90-P). The tensile strength of this composition (4.49 MPa) was only slightly higher than the value determined for PP/GTR90 and clearly differed from the trend line determined for the *Rm* of PP/GTR-P compounds with a filling equal to 30 wt.% to 80 wt.%. This effect is most likely caused by an insufficient amount of the matrix allowing for its effective surrounding of the filler particles.

The effect of higher adhesion between components in PP/GTR compounds is also clearly visible in the graph showing changes in the value of strain at break depending on the filling degree of the tested compounds (Figure 15b). The introduction of GTR to the unmodified copolymer matrix resulted in a significant reduction of the strain at break (*ε_b_*). For example, the average value of *ε_b_* determined for PP was equal to 154%, while in the case of PP/GTR compounds with a filler content between 30 wt.% and 50 wt.%, the strain at the break did not exceed 20%. For compounds containing more than 50 wt.% of the rubber filler, significantly higher *ε_b_* was already observed. For PP/GTR90, a value of approximately 75% was recorded. The modification of the DHBP polypropylene resulted in a reduction of *ε_b_* of the material to 10%. However, all PP/GTR-P compositions with a filling degree ranging from 30 wt.% to 80 wt.% had higher strain at break values both compared to PP-P and also compared to the PP/GTR series compounds of the same filling. For example, the strain at break of the PP/GTR30 was approximately 25%, whereas for the PP/GTR80 composition a value of around 85% was recorded. It is worth noting that increasing the filler content to 90% (PP/GTR90-P) resulted in a reduction of *ε_b_* to 75%, i.e., the value that was also determined for the PP/GTR90. As previously described, this effect is most likely due to an insufficient amount of thermoplastic matrix.

GTR dispersed in the copolymer matrix also changes the impact strength of the obtained materials (Table 5). Unfilled PP was not broken in the impact test carried out at 23 °C. The PP/GTR30 broke due to the impact of the pendulum, and the recorded impact strength value was approximately 24 kJ/m^2^. The observed breaking effect is often found in materials modified with powder fillers and fibers. This phenomenon is due to the poor adhesion between the matrix and the filler particles. Increasing the content of the elastic phase in the material resulted in an increase in the impact strength to 34 kJ/m^2^ (PP/GTR60). Samples with higher fillings were not broken during the impact test. This effect is due to the dominance of the elastic phase in the material, which is characterized by a lower tensile modulus with increasing GTR content. Moreover, the higher impact strength of compounds with a rubber content up to 50 wt.%. is correlated with an increase in the value of the elongation at break. It indicates an increase in the cohesiveness of the compound, which is most likely caused by the decreasing thickness of the rigid thermoplastic layer surrounding the individual GTR particles. Similar effects were observed in the case of compounds modified with organic peroxide. It is worth noting, that the samples did not break from 50 wt.% of GTR content in the material. The results of the Charpy impact strength tests of the PP/GTR samples modified with peroxide also confirm the increase in mechanical adhesion between both phases, despite the fact that many samples were not broken during the test. The increase in the impact strength of compounds as a result of the DHBP use and the increased content of GTR in the material was also observed by Wagenknecht [39] in the impact tensile tests.

**Figure 16:**
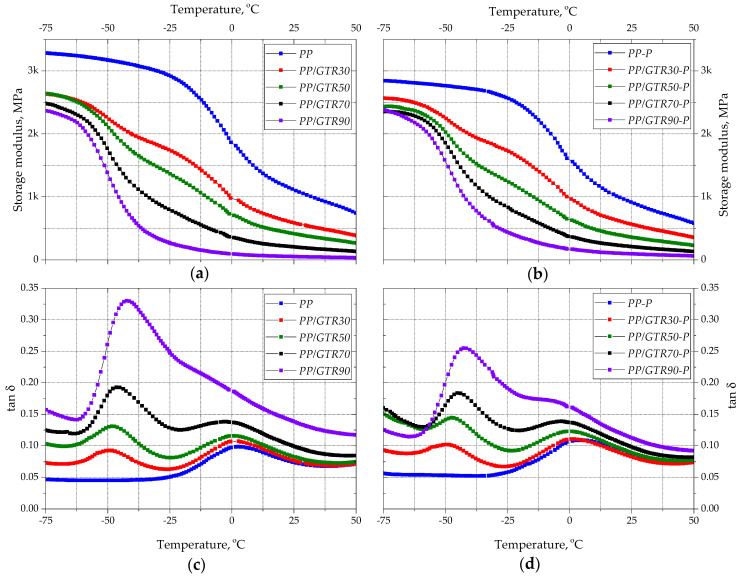
Viscoelastic properties of PP/GTR and PP/GTR-P compounds: storage modulus (**a**,**b**) and mechanical loss factor (**c**,**d**).

The results of the viscoelastic properties research are shown in Figure 16a–d. Along with the increase in GTR content, a decrease in the value of the storage modulus (*E*′) of PP/GTR compounds was observed in the entire temperature range (16a). The step-change of *E*′ associated with the glass transition of unfilled PP was recorded in the range from −25 to 10 °C. In the case of PP/GTR90, a step change of the storage modulus took place in the range of −60 to −40 °C. The clear shift in the glass transition temperature results from the high filling degree of the material, which, despite the presence of PP, has properties similar to those of rubber. Two-step changes of this parameter are visible on the curves showing the changes of *E*′ in compounds with a lower filling degree, which corresponds to the glass transition temperature of rubber and PP. Two clear glass transitions in samples with a lower filling degree are also visible in Figure 16c, which shows changes in the value of the mechanical loss factor (tan δ) as a function of temperature. This indicates the presence of a separately rigid thermoplastic and dispersed elastomeric phase. Moreover, the recorded values of tan δ indicate that an increase in the GTR content in the compound results in an increase in the material’s ability to damp vibrations; it should be emphasized, however, that the largest increase in the value of tan δ was recorded between compounds with 70 and 90 wt.% GTR content. Selective cutting of the PP chains resulted in a decrease in the *E*′ value of unfilled PP and low fill samples. In the case of compounds with a high degree of filling, a reduction in the ability to damp vibrations was observed compared to unfilled compounds with the same GTR content (Figure 16d). The results of the research on viscoelastic properties and the previously described results of rheological tests indicate that the effect of combining a polypropylene copolymer with vulcanized elastomer particles is to obtain a material with thermoplastic properties and high damping. It can be concluded that highly filled PP/GTR materials are characterized by properties similar to thermoplastic elastomers.

### 3.5. Morphology

**Figure 17:**
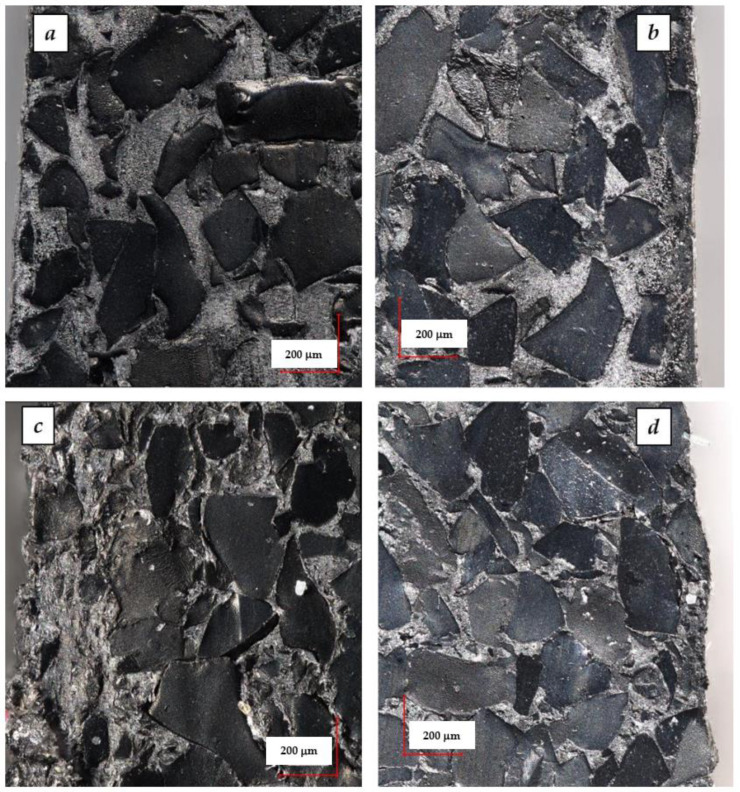
Photography of PP/GTR composites: PP/GTR70 (**a**), PP/GTR70-P (**b**), PP/GTR90 (**c**), PP/GTR90-P (**d**).

Figure 17a–d shows photographs of cross-sections of highly GTR filled polypropylene composites (70 wt.% and 90 wt.%), including their side edges. The photos confirm that despite the very high content of GTR, the samples are characterized by structure coherence. Moreover, all the photos show rubber grains with a size of 400–500 µm, which indicates that during the processing, the GTR was not extensively disintegrated in the plasticizing units of the extruder and injection molding machine. Moreover, it was observed that the outer layer of the samples was dominated by smaller grains with a size not exceeding 200 µm, while large grains are concentrated in the core of the moldings.

**Figure 18:**
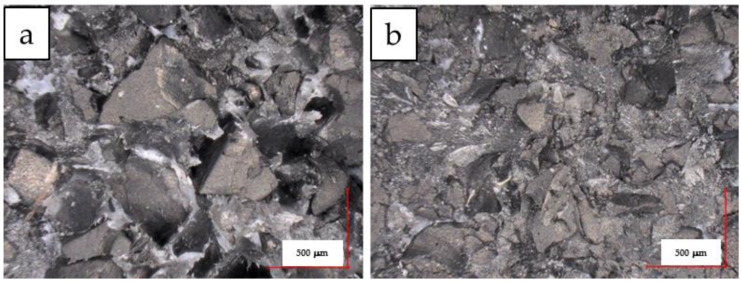
Photography of a fracture test (tensile test) of PP-GTR moldings: PP/GTR50 (**a**), PP/GTR50-P (**b**).

Figure 18 shows photographs of the fracture of PP/GTR50 and PP/GTR50-5 samples obtained during tensile testing. In Figure 18a, ductile deformations of PP, as well as GTR grains not surrounded by a thermoplastic matrix, are clearly visible. In addition, there are visible losses of single grains that were pulled out during fractures. This indicates relatively weak interfacial interactions between the matrix and the rubber particles. A similar effect was observed by Lima et al. for PP/EPR compounds [27]. However, such effects were not observed for the PP/GTR50-P sample (Figure 18b). This indicates greater adhesion between the matrix and the rubber filler in compounds processed with the DHBP. The increase in adhesion explains the significantly greater elongation at the break of the samples with the organic peroxide compared to the samples with the same GTR content that were not modified (Figure 18b). The registered change is accompanied by a simultaneous increase in *Rm*, which confirms the increase in adhesion between the components.

## 4. Conclusions

Highly filled PP/GTR compounds with a filler content of up to 90 wt.% can be manufactured using the fine fraction of ground tire rubber as a filler, with an average grain size of about 400 mm. Properly prepared rubber powder allows for the manufacturing of compounds in standard injection molding machines for thermoplastics. The PP/GTR90 and PP/GTR90-P compositions, despite their very high filling, are characterized by a positive cohesion. The copolymer matrix in the highly filled compounds acts as an adhesive that effectively bonds the individual GTR grains. The effect of more thoroughly surrounding the GTR grains with a matrix of lower viscosity was found. Therefore, DHBP-modified compounds were characterized by higher tensile strength, strain at break and impact strength of highly-filled compositions compared to unmodified materials with the same filler content. Based on the FTIR results obtained, no effect of the DHBP content on the additional chemical bonding processes between the PP matrix and GTR grains was observed. However, it should be emphasized that the observed changes in mechanical properties are correlated with changes in the viscosity of the PP matrix. The registered rheological properties of PP/GTR compounds allow for the production of thick-walled molded parts from this type of material by standard injection molding. Based on the results, an increase in GTR content means the mechanical properties of PP/GTR moldings will worsen but will be acceptable in some mass-market applications. A positive feature is that the GTR content in the PP matrix reduces the shrinkage of moldings and allows for a significant reduction in its cooling time, which shortens the cycle time.

## Figures and Tables

**Table 1:** List of prepared PP/GTR compounds.

Series	Signature	Content (wt.%)		
		PP	GTR	Organic Peroxide
1 (PP/GTR)	PP	100	0	0
	PP/GTR30	70	30	0
	PP/GTR40	60	40	0
	PP/GTR50	50	50	0
	PP/GTR60	40	60	0
	PP/GTR70	30	70	0
	PP/GTR80	20	80	0
	PP/GTR90	10	90	0
2 (PP/GTR-P)	PP-P	98.5	0	1.5
	PP/GTR30-P	68.5	30	1.5
	PP/GTR40-P	58.5	40	1.5
	PP/GTR50-P	48.5	50	1.5
	PP/GTR60-P	38.5	60	1.5
	PP/GTR70-P	28.5	70	1.5
	PP/GTR80-P	18.8	80	1.5
	PP/GTR90-P	8.5	90	1.5

**Table 2:** Rheological parameters of PP/GTR compounds recorded during injection molding.

Samples	Share Stress (Pa)	Share Rate (1/s)	Samples	Share Stress (Pa)	Share Rate (1/s)
PP	84,355	1221	PP-P	16,008	1266
PP/GTR30	164,270	1438	PP/GTR30-P	165,630	1575
PP/GTR50	191,159	1456	PP/GTR50-P	231,241	1648
PP/GTR70	254,190	1553	PP/GTR70-P	283,768	1655
PP/GTR90	378,003	1567	PP/GTR90-P	414,083	1486

**Table 3:** Values of injection pressure and pressure in the mold cavity during filling phase.

GTR Content, wt.%	Injection Pressure, MPa		Pressure in Mold Cavity, MPa	
	PP/GTR	PP/GTR-P	PP/GTR	PP/GTR-P
0	36.9	8.0	18.7	20.5
30	44.6	54.3	24.0	44.8
50	51.3	66.2	23.9	48.9
70	62.4	79.9	31.9	51.8
90	94.0	117.7	42.5	41.5

**Table 4:** Results of calorimetric measurements.

Sample	*T_c_*, °C	*T_m_*, °C	Determined Content of GTR, wt.%
PP	119.5	150.0	-
PP/GTR30	107.6	144.9	33.96
PP/GTR40	107.1	145.1	43.51
PP/GTR50	107.3	144.9	47.37
PP/GTR60	106.6	145.5	59.44
PP/GTR70	105.5	145.6	71.54
PP/GTR80	105.4	144.5	78.60
PP/GTR90	105.2	143.2	89.41
PP-P	119.0	146.0	-
PP/GTR30-P	103.1	139.8	30.64
PP/GTR40-P	103.3	139.9	40.51
PP/GTR50-P	104.5	140.5	51.42
PP/GTR60-P	105.7	141.2	61.56
PP/GTR70-P	104.7	142.0	70.84
PP/GTR80-P	105.3	140.8	79.35
PP/GTR90-P	106.3	142.2	89.68

**Table 5:** Influence of GTR content on Charpy impact strength of PP/GTR compounds.

Filler Content (wt.%)	Charpy Impact Strength (kJ/m^2^)	
	Temperature 23 °C	
	PP/GTR	PP/GTR-P
0	nb	nb
30	23.8	24.8
40	25.3	34.3
50	27.8	nb
60	34.0	nb
70	nb	nb
80	nb	nb
90	nb	nb

## Data Availability

The data presented in this study are available on request from the corresponding author.

## References

[1] Kościuszko A., Sterzyński T., Piszczek K. (2018). Multilayer hybrid polypropylene composite with single and wood-polymer composites layers. Polimery.

[2] Kościuszko A., Czyżewski P., Wajer Ł., Ościak A., Bieliński M. (2020). Properties of polypropylene composites filled with microsilica waste. Polimery.

[3] Barczewski M., Andrzejewski J., Majchrowski R., Dobrzycki K., Formela K. (2021). Mechanical Properties, Microstructure and Surface Quality of Polypropylene Green Composites as a Function of Sunflower Husk Waste Filler Particle Size and Content. J. Renew. Mater..

[4] Gozdecki C., Wilczyński A., Kociszewski M., Zajchowski S. (2015). Properties of wood–plastic composites made of milled particleboard and polypropylene. Eur. J. Wood Wood Prod..

[5] Lewandowski K., Piszczek K., Zajchowski S., Mirowski J. (2016). Rheological properties of wood polymer composites at high shear rates. Polym. Test..

[6] Barczewski M., Mysiukiewicz O., Lewandowski K., Daniel Nowak D., Matykiewicz D., Andrzejewski J., Skórczewska K., Piasecki A. (2020). Effect of Basalt Powder Surface Treatments on Mechanical and Processing Properties of Polylactide-Based Composites. Materials.

[7] Ryu Y., Sohn J.S., Kweon B.C., Cha S.W. (2019). Shrinkage Optimization in Talc- and Glass-Fiber-Reinforced Polypropylene Composites. Materials.

[8] Kościuszko A., Marciniak D., Sykutera D. (2021). Post-Processing time dependence of shrinkage and mechanical properties of injection-molded polypropylene. Materials.

[9] Bazan P., Nosal P., Kozub B., Kuciel S. (2020). Biobased Polyethylene Hybrid Composites with Natural Fiber: Mechanical, Thermal Properties, and Micromechanics. Materials.

[10] Pokorný J., Šál J., Ševčík R. (2021). The Role of Processing Procedures on Properties of Waste Tires Recycled Products. AIP Conf. Proc..

[11] Formela K. (2021). Sustainable development of waste tires recycling technologies—Recent advances, challenges and future trends. Adv. Ind. Eng. Polym. Res..

[12] Hoyer S., Kroll L., Sykutera D. (2020). Technology comparison for the production of fine rubber powder from end of life tyres. Procedia Manuf..

[13] Tamayo A., Rubio F., Pérez-Aparicio R., Saiz-Rodríguez L., Rubio J. (2021). Preparation and properties of sustainable brake pads with recycled end-of-life tire rubber particles. Polymers.

[14] Kosmela P., Olszewski A., Zedler Ł., Burger P., Formela K., Hejna A. (2021). Ground tire rubber filled flexible polyurethane foam—effect of waste rubber treatment on composite performance. Materials.

[15] Araujo-Morera J., Verdugo-Manzanares R., González S., Verdejo R., Lopez-Manchado M.A., Santana M.H. (2021). On the use of mechano-chemically modified ground tire rubber (GTR) as recycled and sustainable filler in styrene-butadiene rubber (SBR) composites. J. Compos. Sci..

[16] Karger-Kocsis J., Mészáros L., Bárány T. (2013). Ground tyre rubber (GTR) in thermoplastics, thermosets, and rubbers. J. Mater. Sci..

[17] Serdar M., Baričević A., Bjegović D., Lakušić S. (2014). Possibilities of use of products from waste tyre recycling in concrete industry. J. Appl. Eng. Sci..

[18] Serdar M., Baričević A., Rukavina M.J., Pezer M., Bjegović D., Štirmer N. (2015). Shrinkage behaviour of fibre reinforced concrete with recycled tyre polymer fibres. Int. J. Polym. Sci..

[19] Yao H., Zhou S., Wang S. (2016). Structural evolution of recycled tire rubber in asphalt. J. Appl. Polym. Sci..

[20] Fernández-Ruiz R., Redrejo M.J., Pérez-Aparicio R., Saiz-Rodríguez L. (2020). Quantification of recycled rubber content of end-of-life tyres in asphalt bitumen by total-reflection X-ray fluorescence spectrometry, Spectrochim. Acta Part B At. Spectrosc..

[21] Alkadi F., Lee J., Yeo J.S., Hwang S.H., Choi J.W. (2019). 3D Printing of Ground Tire Rubber Composites. Int. J. Precis. Eng. Manuf. Green Technol..

[22] Dou Y., Rodrigue D. (2018). Rotomolding of Foamed and Unfoamed GTR-LLDPE Blends: Mechanical, Morphological and Physical Properties. Cell. Polym..

[23] Kakroodi A.R., Rodrigue D. (2013). Highly filled thermoplastic elastomers from ground tire rubber, maleated polyethylene and high density polyethylene. Plast. Rubber Compos..

[24] Mujal-Rosas R., Marin-Genesca M., Ballart-Prunell J. (2015). Dielectric properties of various polymers (PVC, EVA, HDPE, and PP) reinforced with ground tire rubber (GTR). Sci. Eng. Compos. Mater..

[25] Hrdlička Z., Trnka T., Čadek D., Kadeřábková A., Kuta A. (2018). Thermoplastic blends based on waste tyre rubber and polyamide 12. KGK Kautsch. Gummi Kunstst..

[26] Lu Y., Yang Y., Xiao P., Feng Y., Liu L., Tian M., Li X., Zhang L. (2017). Effect of interfacial enhancing on morphology, and rheological properties of polypropylene-ground tire rubber powder blend. J. Appl. Polym. Sci..

[27] Lima P.S., Oliveira J.M., Costa V.A.F. (2015). Partial replacement of EPR by GTR in highly flowable PP/EPR blends: Effects on morphology and mechanical properties. J. Appl. Polym. Sci..

[28] Lima P.S., Oliveira J.M., Costa V.A.F. (2015). Crystallization kinetics of thermoplastic elastomeric blends based on ground tyre rubber. J. Appl. Polym. Sci..

[29] Basso A., Zhang Y., Linnemann L., Hansen H.N. (2021). Study of the distribution of rubber particles in ground tire rubber/polypropylene blends. Mater. Today Proc..

[30] Egodage S.M., Harper J.F., Walpalage S. (2009). Ground Tyre Rubber/Waste Polypropylene Blends—Effect of Composition on Mechanical Properties. Prog. Rubber Plast. Recycl. Technol..

[31] Mujal-Rosas R., Orrit-Prat J., Ramis-Juan X., Marin-Genesca M., Rahhali A. (2012). Study on dielectric, mechanical and thermal properties of polypropylene (PP) composites with ground tyre rubber (GTR). Polym. Polym. Compos..

[32] Majewska-Laks K., Sykutera D., Osciak A. (2021). Reuse of ground tire rubber (GTR) as a filler of TPE matrix. MATEC Web Conf..

[33] Lima P., da Silva S.P.M., Oliveira J., Costa V. (2015). Rheological properties of ground tyre rubber based thermoplastics elastomeric blends. Polym. Test..

[34] Da Silva L.P., Rocha J.S., Pacheco E.B.V., Boucas T. (2008). Mechanical and morphological properties of polypropylene and regenerated tire-rubber blends. Int. J. Polym. Mater. Polym. Biomater..

[35] Elenien K.F.A., Abdel-Wahab A., El Gamsy R., Abdellatif M.H. (2018). Assessment of the properties of PP composite with addition of recycled tire rubber. Ain Shams Eng. J..

[36] Hernández Gámez José F., Hernández Ernesto H., Narro Céspedes Rosa I., Neira Velázquez María G., Solís Rosales Silvia G., Soriano Corral F., González Morones P., Fernández Tavizón S., Díaz de Leon R., Farías Cepeda L. (2016). Mechanical reinforcement of thermoplastic vulcanizates using ground tyre rubber modified with sulfuric acid. Polym. Compos..

[37] Hernandez E.H., Gamez J.F.H., Cepeda L.F., Munoz E.J.C., Corral F.S., Rosales S.G.S., Velazquez G.N., Morones P.G., Martinez D.I.S. (2017). Sulfuric acid treatment of ground tire rubber and its effect on the mechanical and thermal properties of polypropylene composites. J. Appl. Polym. Sci..

[38] Kim S., Lee M., Lee H., Jeong H., Park Y., Jhee K.-H., Bang D. (2016). Effects of peroxides on the properties of reclaimed polypropylene/waste ground rubber tire composites prepared by twin screw extrusion. Elastomers Compos..

[39] Wagenknecht U., Wiessner S., Heinrich G., Michael H., Zichner M. (2006). Effects of interface reactions in compatibilised ground tyre rubber polypropylene elastomeric alloys. Plast. Rubber Compos..

[40] Coutinho F.M.B., Costa T.H.S. (1994). Controlled degradation of polypropylene in solution by organic peroxide. Polym. Test..

[41] Konieczka R., Kaluzny W., Sykutera D. (1997). Fine rubber grinding by rotational cutting. KGK-Kautsch. Gummi Kunstst..

[42] Berzin F., Vergnes B., Delmare L. (2001). Rheological behavior of controlled-rheology polypropylenes obtained by peroxide-promoted degradation during extrusion: Comparison between homopolimer and copolymer. J. Appl. Polym. Sci..

[43] Herlambang B., Bramantika S. (2019). Controlling polypropylene rheological properties by promoting organic peroxide during extrusion with improved properties for automotive applications. J. Phys. Conf. Ser..

[44] Sykutera D., Czyżewski P., Kościuszko A., Szewczykowski P., Wajer L., Bieliński M. (2018). Monitoring of the injection and holding phases by using a modular injection mold. J. Polym. Eng..

[45] Sykutera D., Czyżewski P., Szewczykowski P. (2020). The Microcellular Structure of Injection Molded 686 Thick-Walled Parts as Observed by In-Line Monitoring. Materials.

[46] Yoon L.K., Choi C.H., Kim B.K. (1995). Reactive extrusion of PP/natural rubber blends. J. Appl. Polym. Sci..

[47] Rooj S., Basak G.C., Maji P.K., Bhowmick A.K. (2011). New route for devulcanization of natural rubber and the properties of devulcanizated rubber. J. Polym. Environ..

